# Increasing questionnaire response: evidence from a nested RCT within a longitudinal birth cohort study

**DOI:** 10.1186/s12874-020-01034-7

**Published:** 2020-06-22

**Authors:** Michaela Goodwin, Tanya Walsh, William Whittaker, Richard Emsley, Matt Sutton, Martin Tickle, Michael P. Kelly, Iain A. Pretty

**Affiliations:** 1 The Dental Health Unit, Division of Dentistry, Williams House, University of Manchester, Manchester Science Park, Manchester, M15 6SE UK; 2 Division of Population Health, Health Services Research and Primary Care, University of Manchester, Oxford Road, Manchester, M13 9PL UK; 3 Department of Biostatistics and Health Informatics, Institute of Psychiatry, Psychology and Neuroscience, King’s College London, De Crispigny Park, London, SE5 8AF UK; 4 Primary Care Unit, Institute of Public Health, University of Cambridge, Cambridge, CB2 0SR UK

**Keywords:** RCT, questionnaires, response rates, non-response bias, deprivation

## Abstract

**Background:**

High response rates are essential when questionnaires are used within research, as representativeness can affect the validity of studies and the ability to generalise the findings to a wider population.

The study aimed to measure the response rate to questionnaires from a large longitudinal epidemiological study and sought to determine if any changes made throughout data collection had a positive impact on the response to questionnaires and addressed any imbalance in response rates by participants’ levels of deprivation.

**Methods:**

Data were taken from a prospective, comparative study, designed to examine the effects of the reintroduction of water fluoridation on children’s oral health over a five-year period. Response rates were analysed for the first year of data collection. During this year changes were made to the questionnaire layout and cover letter to attempt to increase response rates. Additionally a nested randomised control trial compared the effect on response rates of three different reminders to complete questionnaires.

**Results:**

Data were available for 1824 individuals. Sending the complete questionnaire again to non-responders resulted in the highest level of response (25%). A telephone call to participants was the only method that appeared to address the imbalance in deprivation, with a mean difference in deprivation score of 2.65 (95% CI -15.50 to 10.20) between the responders and non-responders.

**Conclusions:**

Initially, low response rates were recorded within this large, longitudinal study giving rise to concerns about non-response bias. Resending the entire questionnaire again was the most effective way of reminding participants to complete the questionnaire. As this is a less labour intensive method than for example, calling participants, more time can then be spent targeting groups who are underrepresented. In order to address these biases, data can be weighted in order to draw conclusions about the population.

## Background

There are a number of factors that could be considered in order to maximise questionnaire response rates. First, the mode of administration can be altered, with questionnaires being returned via the post, completed online or through personal interviews. Secondly, the design of the questionnaire could change i.e. length, content or appearance [1]. Thirdly, incentive based approaches could be used to increase response (money, gifts or prize draws) [2]. Finally, the non-response behaviour of the participant could be addressed using a theory-based behaviour change intervention [3]. It is also important to recognise that response is influenced by factors such as age, sex, being a member of a minority group and deprivation, this has been observed throughout numerous cohort studies [4, 5]. Even if overall survey response is increased within a study, these factors may still have such a strong impact that bias remains for particular sociodemographic variables.

### Mode of administration

Postal distribution has been traditionally used for questionnaire studies; however these are associated with a number of issues including printing and postage costs and an inability to use branching or programming to support a participant as they proceed through a questionnaire.

Online surveys, which are typically accessed through a link sent via email, leading respondents to a webpage to complete the survey, have some advantages over the postal method described above. They are cheaper and can, to some extent, be automated using branching logic which allows them to be completed more quickly. However, they also come with a number of limitations including bias with regards to who will respond. Evidence suggests there is uneven access to this type of technology across different populations [6]. Some studies have shown a lower response rate of 10-11% for online questionnaires compared to postal questionnaire [7] with email contact being seen as impersonal [8].

Personal interviews conducted either face-to-face or over the phone can be structured yet flexible, with participants able to seek clarification to questions and discuss why they are being asked. As they are based on personal interaction, participants may be more likely to complete the questionnaire in its entirety if contacted (although actually contacting participants at a convenient time can be problematic) [9]. A limitation to these types of surveys is they are subject to interviewer and responder bias, are expensive and time consuming for the researcher with a potentially lower response or different response across subgroups i.e. working or not working if participants cannot be contacted [10, 11].

### Questionnaire design

There are a variety of elements relevant in questionnaire design to increase response rates. From the way questions are phrased to the layout and appearance of a questionnaire [9]. Edwards [1, 8] conducted a systematic review which indicated researchers increased the response rate by; using coloured ink, having a user friendly layout, a shorter questionnaire and making the questionnaire and letter more personalised.

### Identifying incentives and barriers

Edwards et al [1] noted in their systematic review that incorporating an incentive and including stamped addressed envelopes increased response rates. All types of incentive (monetary or non-monetary) increased response rates but, with an odds ratio of 2.02 (95% CI 1.79 to 2.27), the monetary incentive vs. no incentive provided the largest pooled response effect (49 studies with 46,474 participants). Other strategies, such as following up with an additional contact or sending a second copy of the questionnaire, were also found to significantly and positively influence response rate.

### Theories in behaviour change in non-responders

While the concepts described above detail the simple elements that can facilitate or incentivise participants, an attempt to address non-response can also be made through targeting the behaviour of participants using theoretically informed interventions. Cane *et al.* [12] developed a theoretical domains framework (TDF) that encompasses component constructs used to guide behaviour change interventions. These include the domains of knowledge, skills, social role/identity, beliefs about capabilities and consequences, social influences and motivation [12]. Elements from these can be used to influence the behaviour researchers wished to change (in this case responding to a questionnaire). This can be achieved by incorporating aspects of these domains into information provided to participants, for example as part of a cover letter or leaflet. Table 1 gives an example of this; including the breakdown of the Theoretical Domains Framework described by Cane *et al.,* [12], and each of the behaviour change constructs and definitions in relation to questionnaire response. This breakdown is also based on previous work carried out by Duncan (2015) [3] who used these theoretical domains to increase response rate in a trial in primary care dentistry.

**Table 1:** Behaviour change constructs and descriptions relevant to questionnaire response. (Based on Cane et al (2012) [12] and on Duncan’s et al (2015) [3]

Theoretical domain that could be targeted	Theoretical domain constructs
**Intentions and goals**	**Establish the*intention*to return the questionnaire** Detail original consent and encourage return of questionnaire as soon as possible
	**State the*goals*in relation to returning the questionnaire** Target both immediate goals (completing questionnaire) and longer term goals
**Social influences**	**Relate the research to*social norms*or*conformity*** Provide information on the number of people taking part and completing the questionnaire if appzropriate
**Beliefs about consequences**	**Information of*consequences*and*attitude*** Specific information about the benefits of taking part and what the consequences will be if people do not complete the information. Detail what the expectations of the outcomes will be
**Behavioural regulation**	**Implementation*intention*and how will they put a plan into*action (action planning)*** Detail an example of where, when, and how behaviour will be performed.
**Environmental context and behaviour**	**Identify*barriers*and how these can be overcome** Introduce or define environmental or social stimulus with the purpose of prompting or cueing the behaviour.
**Knowledge**	***Information*about the study, disease and why this is important**

It is vital to understand response and any potential bias as these can affect the validity of studies and the ability to generalise the findings to a wider population. Particularly if the response is skewed in a way that could directly affect the main outcome.

## Methods

### Aims

This paper assesses the changes made throughout baseline data collection on response rates in a longitudinal cohort study. The changes included introducing behavioural change components into a cover letter, altering the layout of the questionnaire and testing the effectiveness of three different participant reminders to complete questionnaires.

A secondary aim was to assess the impact of deprivation on response rates and determine if any changes to the questionnaire or reminders addressed any imbalances. This was included as the outcome of interest (development of dental caries) and has previously been associated with levels of socio-economic deprivation. Given that previous studies have also shown deprivation being associated with response rates, it is important to understand whether response is associated with deprivation and if so whether any of the changes made are able to address any imbalance.

These changes and the introduction of new methods were implemented in a pragmatic fashion within the overall longitudinal study and were deemed warranted given the lower than expected response rates as the study proceeded.

### Study hypothesis

The study’s main null hypotheses were:

- There is no association between levels of deprivation and the response rate.
- There is no difference in response rates according to the nature of the cover letter or methods of sending reminders.
- There is no difference in deprivation-related differences in response rates according to the nature of the cover letter or methods of sending reminders.

### Study design

The data was taken from a prospective comparative study, designed to examine the effects of the reintroduction of water fluoridation, on young children's oral and general health. The details of this study can be found in the published protocol [13].

For this part of the study baseline questionnaires were sent out to consented parents within 12 months of the birth of their child. During these 12 months changes were made every 4 months to increase the questionnaire response rate. The changes were submitted and approved by NRES Committee East of England - Cambridge South**.** The changes made are described below and summarised in Table 2

**Table 2:** Changes made in each wave of the study

Wave	Time period	Description of questionnaire distribution		Changes between waves
		1^st^ attempt	2^nd^ attempt	
Wave 1	4 months	Questionnaire and cover letter (V1) sent via email or post (stamped addressed envelope included)	Questionnaire resent via post	-
Wave 2	4 months	Questionnaire layout updated and cover letter (V2), sent via email or post (stamped addressed envelope included)	Questionnaire resent via post	Cover letter updated to utilise behaviour change techniques designed to increase response, free pen included, questionnaire updated
Wave 3	4 months	Questionnaire and cover letter (V2), sent via email or post (stamped addressed envelope included)	Reminder by: Postcard Telephone call Questionnaire resent	RCT conducted for those who did not complete the questionnaire on the first send out to determine if one reminder was more effective or reduced bias

For the first 4 months participants received a questionnaire with a standard cover letter by email or by post (this period is referred to as Wave 1).

During the following 4 months participants received a questionnaire with an amended layout to make it easier to read. In addition an updated cover letter was used which utilised simple behaviour change techniques to encourage response such as motivation and goal setting, beliefs about the consequences of completing the questionnaire, action planning and further information on the study. The updated cover letter and questionnaire was sent along with a free pen (this period is referred to as Wave 2).

For the last 4 months a nested RCT was used to determine which three methods to remind non-responders to return their questionnaire was most effective. The three methods were reminders; by phone, by postcard or sending the whole questionnaire again for those who did not reply to the first questionnaire. In order to determine which were more effective, the methods of addressing non-response were randomly allocated to families who didn’t respond to the first questionnaire (this period is referred to as Wave 3).

It should be noted if randomisation was not possible for wave 3 (i.e. if no phone number was held for a participant and they had been allocated a telephone call they were assigned one of the other reminders). A de-identified list of participants (those who did not respond to the initial questionnaire) was created each month along with a computer generated randomisation sequence and allocated to each unique ID number for postcard, telephone or repeat questionnaire reminder on a 1:1 without stratification. Participants would be aware of their status (whether or not they received a telephone call) but were unaware of any other groups status and that this was monitored as part of a RCT. It was impractical for the researcher administering the intervention (contacting people by telephone to complete the questionnaire, etc) and recording the outcome (completing the questionnaire) to be blinded to the group status.

### Variables of interest

Data were gained from information recorded during the consent process or from questionnaires. The Index of Multiple Deprivation (IMD) was obtained based on the individual’s postcode of residence. IMD quintiles were arrived at using the National Perinatal Epidemiological Unit (NPEU) IMD using the breakdown given in Appendix, with a higher IMD score indicating a more deprived area [14]. In this study we used IMD to measure the deprivation of the individual’s area of residence. This Index combines data from many sources to characterise and summarise deprivation across many domains.

The primary outcome was the percentage of participants who had returned a questionnaire.

### Study participants

**Fig. 1:**
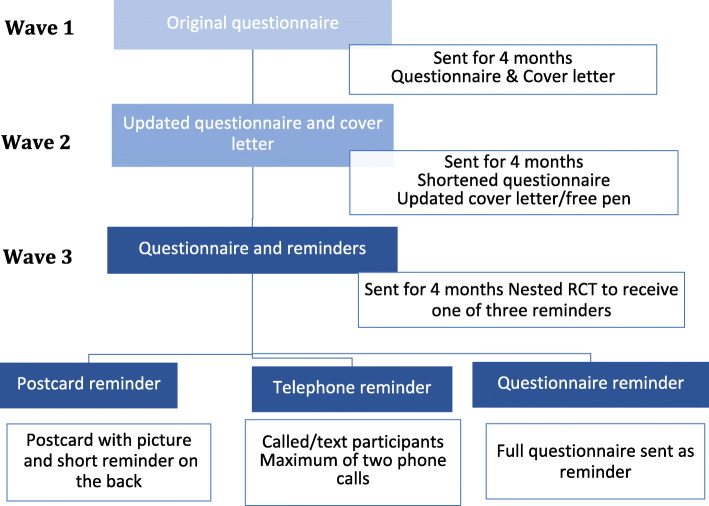
Flow chart of methods used to increase questionnaire response for each wave

Participants eligible for this study were those who had a child born in one of two designated hospitals in Cumbria, United Kingdom, from 1^st^ September 2014 to 31^st^ August 2015. This formed the study population. Groups were subsequently formed from waves based on when they were born, which included; Wave 1 - those who received both the standard questionnaire and cover letter, Wave 2 - an altered questionnaire and cover letter and finally Wave 3 - the third group were randomised to receive one of three different reminders following no response to the questionnaire, further illustrated in Fig 1.

### Statistical analysis

Statistical significance level was set at 5% for all analysis. Statistical analysis was performed using SPSS (IBM Corp. Released 2017. IBM SPSS Statistics for Mac, Version 25.0. Armonk, NY: IBM Corp).

Mean IMD scores were compared between responders and non-responders using an independent two-tailed t-test, after confirming the underlying assumptions were met. Logistic regression analysis was carried out to determine the strength of association between response (yes/no) with type of reminder and levels of IMD (model 1) and the interaction between these (model 2) as predictors. Analysis were performed to determine if the waves of participants who were included over 3 different time periods were significantly different from each other (given the only known difference was the time period questionnaires were distributed over the course of one year).

### Ethical considerations

The water fluoridation study has been reviewed and approved by an NHS ethics committee (14/EE/0108) and NIHR. All participants provide written informed consent prior to enrolling in the study for themselves (parent) and their child.

## Results

Out of the 1824 participants who consented to be part of the study 47% completed the baseline questionnaire for their child. Overall those who responded to the questionnaire had a mean IMD score of 22.3 (95% CI 21.4 to 23.2) with non-responders having a mean of 28.4 (95% CI 27.4 to 29.4), which indicated non responders lived in significantly more deprived areas than responders (t(1701)=9.011, p=0.001).

### Results: Changes to improve response rates

Throughout the year baseline data was collected, two changes were implemented to attempt to increase response rate. This resulted in what has been described as three waves of data collection.

**Fig. 2:**
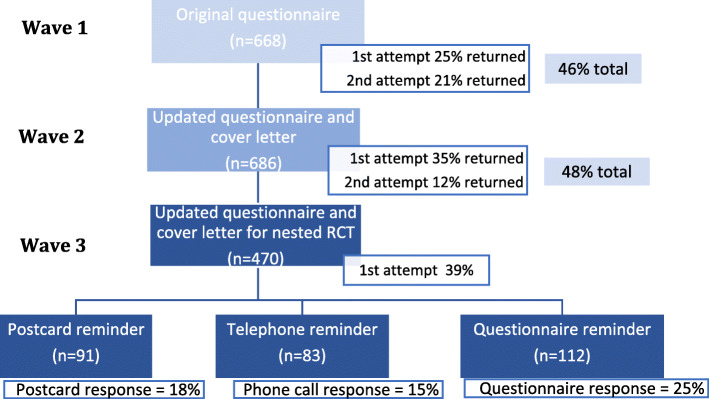
Flow diagram of response for each wave

In Wave 1 non-response was an issue overall (from both email and postal questionnaires) with only 25% (164/668) of questionnaires returned from the first attempt. However a second postal attempt elicited an additional 21% (141/668) of questionnaires returned; providing an overall response rate of 46% (305/668) after 2 attempts (see Fig. 2).

In Wave 2 following the change to the cover letter and additional free pen, response rose from the 25% (164/668) in Wave 1 to 35% (237/686) when looking at response from the initial mail out of questionnaires to this group. However when non responders were sent the questionnaire a second time this elicited fewer returns, resulting in a similar overall response of 48% (329/686) after 2 attempts (see Fig. 2). This indicates the overall response rate had not improved significantly between Wave 1 and Wave 2. This is despite a higher response rate observed for Wave 2 after the first send out compared to the first send out for Wave 1. This is illustrated further in Fig. 2, which shows the proportion of those responding out of those receiving the questionnaire on each attempt.

As response rates only improved marginally between Wave 1 and 2 the research team decided to look at different types of reminders in order to understand if one method would elicit a better response to those who had not initially completed the questionnaire. Results from the random allocation of the three different methods to remind participants to complete the questionnaire showed resending the entire questionnaire was the most effective method in gaining responses. Twenty-five percent of those resent the whole questionnaire as a reminder in the second attempt completed and returned it. This was compared to a postcard reminder, which resulted in 18% (n=91) of questionnaires returned, and phone calls where 15% (n=83) of questionnaires were completed (see Fig. 2).

### Results- response and deprivation

**Fig. 3:**
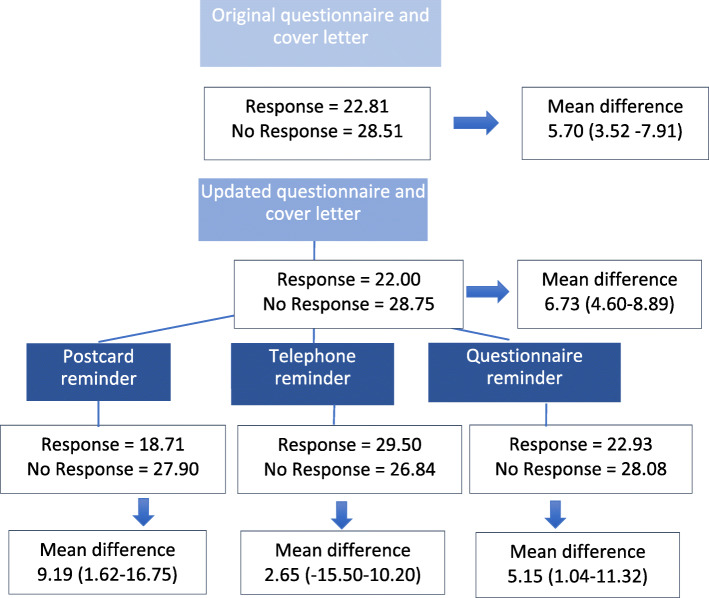
Flow chart showing IMD by response for each wave

Strong evidence of a difference in IMD between those who completed the questionnaire compared to those who didn’t complete in wave 1 was observed (t (613) = 4.986, p = 0.0001, mean difference scores are presented in Fig. 3. This difference was still observed after changes were implemented in wave 2. Therefore, despite a slight increase in response during the initial send out between wave 1 and 2 this increase has not addressed the imbalance of IMD between responders. When exploring the different reminder methods to increase response rate for non-responders, contacting participants over the phone was the only method that appeared to readdress the IMD differences observed, with a mean difference in deprivation score of 2.65 (95% CI -15.50 to 10.20) between the responders and non-responders.

To assess the effect of deprivation and different reminders, a logistic regression was estimated with the variables entered separately (see Table 3- model 1) and as an interaction. Model 1 indicated the odds of a participant completing a questionnaire was 1.99 times higher for those receiving the whole questionnaire again compared to those who received a telephone call (95% CI 0.93 to 4.26). There was a significant effect of deprivation (odds ratio of 0.98 (95% CI 0.96 to 0.99). When interactions with deprivation were included (See Table 4 - model 2) only the postcard vs the telephone reminder showed a significant interaction effect indicating that, in comparison to the telephone reminder, whether a participant responded to the postcard reminder was influenced by the deprivation score of participants.

**Table 3:** Logistic regression for response by reminder and deprivation (Model 1)

Variable	Odds Ratio	Standard Error	*p*-value	95% CI
Resent telephone (ref)	1.00	-	-	-
Resent questionnaire	1.99	0.387	0.074	0.93-4.26
Resent postcard	1.17	0.422	0.705	0.51-2.68
Deprivation score	0.98	0.012	0.042	0.96-0.99

Model *x*^2^ (3) = 8.463 p = 0.037 Nagelkerke R^2^= 0.048 = 4.8% variance explained

**Table 4:** Logistic regression for response by reminder and deprivation (Model 2- interaction)

Variable	Odds Ratio	Standard Error	*p*-value	95% CI
Resent telephone (ref)	1.00	-	-	-
Resent questionnaire	5.77	0.833	0.035	1.13-29.55
Resent postcard	5.49	0.891	0.056	0.96-31.52
Deprivation score	1.01	0.021	0.562	0.97-1.05
Resent telephone * deprivation score				
Resent questionnaire * deprivation score	0.96	0.027	0.137	0.91-1.01
Resent postcard * deprivation score	0.94	0.033	0.051	0.88-1.00

Model *x*^2^ (5) = 13.701 p = 0.026 Nagelkerke R^2^= 0.072 = 7.2% variance explained

### Results – Consent / Response and Deprivation quintiles

Table 5 shows the distributions of the populations who consented and who responded across deprivation quintiles. While a significant proportion of the population is located in more deprived areas (quintiles 4 and 5), a higher proportion of those within the least deprived quintiles responded to the questionnaires compared to the distribution of the population (quintiles 1 and 2).

**Table 5:** Population and sample difference in deprivation quintiles

	Quintile 1 (least deprived)	Quintile 2	Quintile 3	Quintile 4	Quintile 5 (most deprived)
Population	7%	16%	21%	30%	26%
Sample of those consented	8%	17%	22%	29%	24%
Sample of those responding	10%	21%	26%	26%	17%
Difference between responded and population	3%	5%	5%	-4%	-9%

We ran a one way ANOVA to explore if there were any differences in deprivation or age of parent across the three waves to determine if the groups were significantly different from each other given the variables available. The differences for deprivation and age were not statistically significant (F(2,1699) = 0.562, p = 0.570) and (F(2,1803) = 2.724, p = 0.066 respectively.

## Discussion

We noted that throughout the first four months of baseline data collection the response rate was lower than expected. Therefore an ethical amendment was sought in order to change the cover letter using behaviour change techniques identified by Cane and previously implemented in other dental studies [3, 12].

In wave 1 (which utilised a standard letter) 25% of participants responded to the first attempt, which rose to an overall response rate of 46% following a second attempt. When the cover letter was updated, an increase in response rate was observed for the first attempt with 37% of people responding. A smaller number of people responded to the second attempt and therefore an overall response rate following the updated cover letter was 48%. A marginal improvement in response rate was therefore achieved.

When exploring the difference in deprivation of responders before and after the changes were implemented it was apparent that while a small increase in response was observed between wave 1 and 2, this increase did not address the difference in deprivation observed between responders and non-responders.

As response rates to the initial distribution of questionnaires had improved, the research team decided to explore the effectiveness of interventions to increase response to a second administration of the questionnaire (for those who initially hadn’t responded to the questionnaire). The options were to resend the entire questionnaire again, send a reminder postcard or use of telephone call to the participants. Resending the entire questionnaire was the most effective method in increasing response compared to the other approaches used in this study. Telephone calls to participants appears to be the only method that readdressed the imbalance in deprivation between responders and non-responders.

Although these results should be treated with caution they do indicate resending questionnaires could be the most appropriate method to improve response overall and reflects similar RCTs which have demonstrated repeat mailings are one of the most effective ways to increase response rates [15]. Specific groups within the study could then be targeted with phone calls to attempt to address the imbalance in deprivation of those who do not respond. This strategy would be an effective use of time and resource, as calling participants is a more cumbersome method with access to phone numbers not always possible. Particularly given that of the three methods tested, calling participants resulted in the fewest responses overall.

It should be noted even if response rate is improved, it may not be sufficient to reduce non-response bias i.e. a study may see a statistically significant improvement but not a meaningful benefit to the study (this needs to be weighed against the cost of increasing non response). In addition, as previously described, there are other aspects to consider when resending questionnaires or recontacting participants in relation to response rate; these include deprivation, education, gender, ethnicity, and age [5]. An improvement in non-response may still result in a bias in who responds. Therefore these aspects still need to be taken into account when looking at methods to increase response rate.

The reasons given for non-response when contacting participants over the phone were; time constraints, busy or chaotic lifestyles (particularly with a new born baby), or moving home since their address had been recorded. This is another reason why phone calls were important in certain cases as the mobility of the population meant certain participants had not received the questionnaire in the first mailing. Phone calls also appeared to be an appropriate way of administering the questionnaire to some parents as they stated it would be unlikely they would remember to return the written questionnaire through the post.

### Sources of bias

The data were taken from a larger longitudinal survey being conducted over 5 years. The survey employed a census approach, therefore every individual who gave birth in the Cumbria area of England over the period of one year were approached to take part. A sampling strategy was consequently not required to gain a representative sample of the population as this was a whole population study. Despite this study utilising a census approach there are still possible sources of bias in its representativeness to the target population.

Social desirability and approval bias [16] is a limitation throughout most surveys but is difficult to quantify the effect it will have. Parents who wish to be perceived as good, knowledgeable and diligent in relation to raising their child may alter some of their answers if they believed they are more in keeping with social norms and if their behaviours are thought to go against recommended guidance [17, 18]. However there is no reason this social desirability bias should differ across groups if they are balanced at baseline (or significantly impact if an imbalance at baseline is addressed within the analysis). The main impact from social desirability bias is that certain confounders may represent a weaker effect on the outcome.

Recall bias should be less of an issue as, in this longitudinal study, questions pertain to what a parent and child are doing at the time the questionnaire is administered rather than recalling past behaviour. However there was certain information parents struggled to provide, such as height/length of child. Parents noted while some information was collected as part of their child’s regular development (such as weight) by other health professionals, height was not and therefore was only answered by 30% of those responding.

It was noted that responders were significantly different from non-responders when looking at deprivation. As deprivation is associated with the outcome of interest in this longitudinal study (tooth decay) [19] this means the results should be adjusted to account for this [20].

### Study limitations

A limitation in relation to the methods used to increase response rate is that only the reminder section of this research (whether respondents received a reminder by telephone, questionnaire or posted) utilised an RCT to test these methods. In relation to study design, RCTs are conventionally placed near the top of the hierarchy of evidence. As randomly allocating individuals to different interventions helps reduce bias (such as selection bias) by balancing unknown prognostic factors at baseline [21]. However the change to the cover letter was evaluated in a different way by comparing it to the data collected for the population in the last four months there are a number of issues with this method. There could have been something different about the population who responded to the questionnaire in wave 1 to wave 2 to wave 3. For example data collected could have been influenced by additional external factors (i.e. disruption to the postal system) or seasonal effects (e.g. close to Christmas, summer holidays) could have also affected the response rate. No differences in deprivation (IMD) were observed between the groups, the only known difference between them was the date they were born throughout the year. Comparing groups over different periods of time weakens the conclusions that can be drawn from the data collected. It is acknowledged this was done as a pragmatic step and is being reported as such. Therefore caution should be taken in the conclusions drawn from this data in relation to increasing response rates.

### Implications

It can be observed that a non-response bias is present in this data set. The implication for the water fluoridation study (and possible future studies) is to take this into account and adjust for these differences in the analysis and interpretation of the data. There are a variety of ways of weighting data in order to address representativeness in surveys. These include *inverse probability weights* [22] i.e. 1/probability that unit is selected, or to account for non-response, a model of the probability of selection such as logistic regression could to be performed (sometimes called propensity weighting). Weighting can also be achieved adjusting for an auxiliary variable [20]. In this case the auxiliary variable on which we have information for both consent and response samples is deprivation quintiles. As the percentages of people who responded are different from the population, i.e. the population consists of just 7% of people who are labelled as least deprived, yet more people within this range responded to the questionnaire (10%). Therefore this group is over represented in the response. In order to attempt to rectify this a weight can be assigned to each person based on this skew.

Work by Groenwold et al (2012) [23] has discussed options in regards to missing data analysis including; simply omitting participants with any missing data, imputation and the missing indicator method, where missing observations are set at a fixed value for example ‘0’ and a dummy variable is used within an analytical model to indicate if this variable is missing. Each of these methods have pros and cons but for non-randomised studies such as the current longitudinal epidemiological study, certain methods are incompatible. These include omitting participants where missing data occurs or the missing indicator method. In this situation the recommendation is to use multiple imputation [23, 24]. Missing data can have additional complications in longitudinal research as many methods do not take into account that variables may be correlated and relationships exist across time.

## Conclusion

### Addressing non-response bias

Non-response bias is apparent in this study and therefore techniques to minimise this will need to be incorporated in the analysis, weighting the data is an appropriate method in order to reduce the likely effect of this bias and provide results, which more closely represent the population being studied. This is applicable when information is available about non-responders/consenters or about the population as a whole.

### Increasing response rate

There is limited evidence of the methods to improve response rates to postal questionnaire in health research [25]. While some caution should be taken in utilising the results of this data, given the primary outcome and the smaller sample sizes when groups were looked at individually it does indicate repeat mailings offer the most promising method of maximising response. One the other hand, telephone contact may offer the best method to readdress potential differences in deprivation of responders/non-responders. Changing the format/layout of the questionnaire, adding behavioural change techniques to a cover letter and potentially the addition of a free pen may have a small positive effect on response.

## Data Availability

The datasets generated and/or analysed during the current study are not publicly available as the overall study (on water fluoridation) will not be completed until 2021.

## Appendix

**Table 6:** IMD Quintile by IMD score

Quintile group	IMD score range
1	≤ 8.49 (Least deprived)
2	8.5 - 13.79
3	13.8 - 21.35
4	21.36 - 34.17
5	≥ 34.18 (Most deprived)

## Abbreviations

IMD: Index of Multiple Deprivation
NIHR PHR: National Institute for Health Research - Public Health Research
NPEU: National Perinatal Epidemiological Unit
RCT: Randomised Control Trial
TDF: Theoretical Domains Framework

## References

[1] Edwards P, Roberts I, Clarke M, DiGuiseppi C, Pratap S, Wentz R, Kwan I (2002). Increasing response rates to postal questionnaires: systematic review. BMJ..

[2] Fairhurst C, Bell K, Clark L, Mitchell N, Lenaghan E, Blacklock J, Shepstone L, Torgerson D (2015). Scoop pen sub-study-a ‘trial within a trial’ of enclosing a pen in questionnaire mailings to increase response rate. Trials..

[3] Duncan A, Bonetti D, Clarkson J, Ramsay C (2015). Improving trial questionnaire response rates using behaviour change theory. Trials..

[4] T. Mostafa, G. Ploubidis, Millennium Cohort Study - Sixth Survey 2015-2016, 2017. http://doc.ukdataservice.ac.uk/doc/8156/mrdoc/pdf/mcs6_report_on_response.pdf (Accessed 11 Mar 2020).

[5] Corry NH, Williams CS, Battaglia M, McMaster HS, Stander VA (2017). Assessing and adjusting for non-response in the Millennium Cohort Family Study. BMC Med Res Methodol..

[6] Scott A, Jeon S-H, Joyce CM, Humphreys JS, Kalb G, Witt J, Leahy A (2011). A randomised trial and economic evaluation of the effect of response mode on response rate, response bias, and item non-response in a survey of doctors. BMC Med. Res. Methodol..

[7] Shih T-H, Xitao Fan X (2008). Comparing Response Rates from Web and Mail Surveys: A Meta-Analysis. Field Methods..

[8] Edwards PJ, Roberts I, Clarke MJ, Diguiseppi C, Wentz R, Kwan I, Cooper R, Felix LM, Pratap S (2009). Methods to increase response to postal and electronic questionnaires.

[9] McColl E, Jacoby A, Thomas L, Soutter J, Bamford C, Steen N (2001). Design and use of questionnaires:a review of best practice applicable to surveys of health service staff and patients.

[10] Bowling A (2005). Mode of questionnaire administration can have serious effects on data quality. J. Public Health (Bangkok)..

[11] Alreck P, Settle R. Survey Research Handbook. 3rd ed. McGraw Hill, New York; 2004.

[12] Cane J, O’Connor D, Michie S (2012). S Validation of the theoretical domains framework for use in behaviour change and implementation research. Implement. Sci..

[13] M. Goodwin, R. Emsley, M. Kelly, E. Rooney, M. Sutton, M. Tickle, R. Wagstaff, T. Walsh, W. Whittaker, I.A. Pretty, The CATFISH study protocol: an evaluation of a water fluoridation scheme, BMC Oral Health. 16 (2016) 8. doi: 10.1186/s12903-016-0169-0. 10.1186/s12903-016-0169-0 PMC4736087 26831505

[14] National Perinatal Epidemioology Unit, NPEU IMD Tool, Univ. Oxford, Engl. (2016). https://tools.npeu.ox.ac.uk/imd/ (accessed 20 Aug 2016).

[15] A.-M. Glenny, H. V Worthington, K.M. Milsom, E. Rooney, M. Tickle, Strategies for maximizing consent rates for child dental health surveys: a randomised controlled trial. BMC Med Res Methodol. 1 (2013) 108. doi: 10.1186/1471-2288-13-108. 10.1186/1471-2288-13-108 PMC3846683 24006895

[16] Shulman AD, Silverman I (1974). Social Desirability and Need Approval: Some Paradoxical Data and a Conceptual Re-evaluation. Br. J. Soc. Clin. Psychol..

[17] Sanzone LA, Lee JY, Divaris K, DeWalt DA, Baker AD, Vann WF (2013). A cross sectional study examining social desirability bias in caregiver reporting of children’s oral health behaviors. BMC Oral Health..

[18] Fisher RJ, Katz JE (2000). Social-desirability bias and the validity of self-reported values. Psychol. Mark..

[19] McGrady MG, Ellwood RP, Maguire A, Goodwin M, Boothman N, Pretty IA (2012). The association between social deprivation and the prevalence and severity of dental caries and fluorosis in populations with and without water fluoridation. BMC Public Health.

[20] Bethlehem J (2009). Applied Survey Methods.

[21] Evans D (2003). Hierarchy of evidence: a framework for ranking evidence evaluating healthcare interventions. J Clin Nurs.

[22] Horvitz DG, Thompson DJ (1952). A Generalization of Sampling Without Replacement From a Finite Universe. Source J Am Stat Assoc.

[23] Groenwold RHH, White IR, Donders ART, Carpenter JR, Altman DG, Moons KGM (2012). Missing covariate data in clinical research: when and when not to use the missing-indicator method for analysis. Can Med Assoc.J..

[24] Sterne JAC, White IR, Carlin JB, Spratt M, Royston P, Kenward MG, Wood AM, Carpenter JR (2009). Multiple imputation for missing data in epidemiological and clinical research: potential and pitfalls. BMJ.

[25] Nakash RA, Hutton JL, Jørstad-Stein EC, Gates S, Lamb SE (2006). Maximising response to postal questionnaires--a systematic review of randomised trials in health research. BMC Med Res Methodol.

